# Mental health, welfare and state support: Findings from a novel data linkage

**DOI:** 10.23889/ijpds.v8i2.2223

**Published:** 2023-09-14

**Authors:** Sharon Stevelink, Matthew Hotopf, Ira Madan, Ava Phillips, Ray Leal, Nicola T Fear

**Affiliations:** 1 King's College London, London, United Kingdom; 2 Guy's St Thomas NHS Foundation Trust, London, United Kingdom

## Objectives

To describe the process and outcomes of establishing a unique data linkage between mental healthcare records from the South London and Maudsley (SLaM) NHS Foundation Trust with benefits records from the Department for Work and Pensions.

## Method

 448,404 IDs of patients who accessed secondary mental healthcare services at SLaM were sent to the DWP, including personal identifiers. Data from SLaM covered years 2007-2019, whereas data from DWP covered years 2005-2020. A deterministic data linkage approach was used.

## Results

A linkage rate of 93% was achieved. Patient groups who were less likely to be linked were women, those from a racial and ethnic minority background and younger patients. Benefit receipt was high among patients, with 83% of patients having received benefits during the 15-year follow-up period. Benefits most frequently received included unemployment related or income replacing disability benefits.

## Conclusion

This data linkage is the first of its kind in the UK to demonstrate the use of routinely collected mental health and benefits data. It provides opportunities to generate much needed high-quality evidence that can be used to achieve change, investment in services and translation into policy and practice.

